# Bladder cancer: Need for a standardized protocol of management

**DOI:** 10.4103/0970-1591.38593

**Published:** 2008

**Authors:** Nitin S. Kekre

**Affiliations:** Editor, IJU, Department of Urology, Christian Medical College, Vellore - 632 004, Tamilnadu, India. E-mail: editor@indianjurol.com

It certainly appears that the incidence of bladder cancer is increasing in India. There are many issues about bladder cancer that are being debated in international forums. For example, it is now being accepted that the term ‘superficial bladder cancer’ should be replaced with ‘non-muscle invasive bladder cancer’. Most of us agree today that BCG is the best available intravesical chemotherapy for CIS and T1G3, but its exact dose, duration and maintenance protocol remain controversial. Today, we understand that more than 80% of newly diagnosed invasive bladder cancers are invasive from the outset. We also believe that radical cystectomy offers the best opportunity of cure. However, despite declining mortality from radical cystectomy, the overall cancer specific survival has not improved significantly. This is mainly due to our inability to separate organ-confined diseases from non-organ-confined. Most of the imaging modalities available today fail to detect micrometastatic disease in pelvic lymph nodes. Will this improve with the combined use of lymphoscintigraphy and CT (SPECT CT)? Should one perform such a radical operation in the presence of an obvious lymph node disease? What is the precise role of chemotherapy in the management of invasive bladder cancer? Should it be used in neoadjuvant or adjuvant setting? There are many questions that still remain to be answered. Most of the urologists continue to use adjuvant chemotherapy despite the fact that available evidence (level 1) supports the use of neoadjuvant chemotherapy. In such scenario, it is very important to follow uniform guidelines based on good quality evidence. It is important to perform studies in Indian setting and find answers which would be uniformly applicable to our patient population. Dr. Sudhir Rawal and his colleagues have attempted to answer these questions in this symposium on bladder cancer in this issue. I sincerely thank them for their efforts.

This issue also features four review articles. The first three of them cover important urooncological topics. Radioguided surgery is gaining attention in urological cancer. Dr. Weckermann from Augsburg, Germany, has extensively reviewed this subject in the article “Radioguided surgery in urological malignancies”.

Biomarkers have a very important role in the diagnosis and follow-up of diseases, especially cancers. However, a biomarker can be helpful only if we have effective therapy for that disease. RCC, although a common urological cancer, has no acceptable universal biomarker either for diagnosis or follow-up. The future holds promise for the development of effective second-line treatment for advanced RCC, and then there will be a need for a specific biomarker. Dr. Hiroshi Kitamura from Sapporo Medical University, Japan, has reviewed recent developments in this field.

Dr. Pratyush Ranjan and colleagues from AIIMS, New Delhi, have reviewed the role of HIFU and cryotherapy in prostate cancer, which are emerging as alternatives in the management of prostate cancer. Early results were promising in carefully selected patients, but as rightly pointed out these newer modalities will require further scrutiny in comparison to other accepted treatments for early carcinoma prostate. Dr. Vibhash Mishra and colleagues from Slough, UK, provide a very lucid review on a complex topic “chronic prostatitis”, a disease difficult to diagnose and treat, and often leaves the patient in dissatisfaction and the urologist in frustration.

More importantly, this issue features a new column titled “Legends of Indian Urology”. This column would focus on those legendary personalities who have contributed immensely to the science and cause of urology in India and have helped to put the Urological Society of India on the national and international map. I am very pleased that the first article in this column features the legendary Dr. GM Phadke who was the first President of the Association of GU Surgeons of India. Dr. Phadke was a great surgeon, popular teacher and above all a good human being. His work on obstructive azoospermia got him international acclaim. I am sure his life would continue to inspire generations of the present and future. I am thankful to Dr. Anita Patel for writing this feature.

I look forward to see you all at 41^st^ Annual meeting of USI at Chennai. Let me once again wish you all a very happy and rewarding 2008.

